# Attacking tumor cells with a dual ligand for innate immune receptors

**DOI:** 10.18632/oncotarget.484

**Published:** 2012-05-09

**Authors:** Johan Garaude, J. Magarian Blander

**Affiliations:** INSERM U1040, Institut de Recherche en Biothérapie, Montpellier Cedex 5, France; Mount Sinai School of Medicine, Immunology Institute, Department of Medicine, and the Tisch Cancer Institute, New York, NY, USA

Nod-like receptors (NLRs) are being implicated in an increasing number of biological processes including carcinogenesis. Whether these innate immune receptors can be exploited in anti-tumor therapies is still uncertain. We have shown that engineered flagellin-bearing tumor cells trigger NLRs which cooperate with Toll-like receptor 5 (TLR5) to induce robust anti-tumor T cell responses and tumor rejection. These findings demonstrate great potential for dual targeting of TLRs and NLRs in the design of optimal cancer vaccines.

Pathogen-associated molecular patterns (PAMPs) are microbial structures that signify infection to the immune system upon engaging pattern recognition receptors (PRRs). Among PRRs, the NLRs constitute a recently identified family of cytosolic innate immune receptors that have been implicated in various diseases including cancer [1]. Some of these receptors have the ability to initiate the formation of multiprotein complexes, termed inflammasomes, which induce caspase-1 activation to mediate the proteolytic activation of the pro-inflammatory cytokines interleukin (IL)-1β and IL-18, and can initiate a programmed cell death termed pyroptosis. However, a first signal is needed to stimulate transcription of the precursors pro-IL-1β and pro-IL-18, suggesting that some NLRs can only function in co-operation with other signaling pathways [1]. This signal can efficiently be provided by engagement of another family of innate immune receptors, the Toll-like receptors (TLRs), activation of which leads to cytokine production, T cell co-stimulatory molecule expression, and enhanced antigen presentation [1,2]. While ligands for TLRs have been introduced as adjuvants in vaccine compositions and are currently being tested for use in the clinic, direct manipulation of NLR signaling has not been extensively explored. This will certainly change, however, especially with the recent demonstration that some clinically approved adjuvants rely on inflammasomes to mediate their actions [3].

Among NLR ligands, the bacterial protein flagellin is of particular interest since it is recognized by TLR5 and the NLR NAIP5 (neuronal apoptosis inhibitor protein 5), which partners with the NLR NLRC4 (NLR family, CARD domain containing protein 4) in the cytosol [4,5]. Therefore, sensing flagellin by myeloid cells such as macrophages and dendritic cells (DCs) mobilizes signal transduction downstream of two major families of PRRs, a property that can possibly be exploited in immunotherapy. We tested this possibility by introducing flagellin into different tumor cell lines, a strategy that abrogated tumor development upon subcutaneous or intravenous injection of these flagellin-expressing cells, and induced DC-mediated tumor antigen presentation to CD4^+^ and CD8^+^ T cells [6]. When used as a vaccine, irradiated flagellin-bearing tumor cells efficiently protected mice from tumor development after challenge with parental tumor cell lines. Interestingly, mutation of specific leucines in the C-terminal domain of flagellin abrogated NAIP5/NLRC4-mediated inflammasome formation [6,7], restored the ability of flagellin-expressing cells to form tumors *in vivo*, and impaired their anti-tumor vaccine properties. More surprisingly, we found that it also abrogated tumor-specific adaptive T cell responses, suggesting that NLR signaling was important for T cell priming [6]. In addition, abrogation of TLR5 signaling by use of TLR5 deficient mice or the introduction of a mutation in the D1 domain of flagellin, also impaired immune responses demonstrating that flagellin recognition by TLR5 was also required [6]. Our results suggest that it can be possible to include adjuvants that directly target NLRs in future vaccine designs that already incorporate TLR stimulation. However, dual targeting of TLRs with NLRs will likely generate a particular cytokine environment that needs to be carefully assessed in the efforts to ameliorate the quality of anti-tumor immune responses.

Previous work by Blander and Medzhitov uncovered an interesting property of TLR signaling. Ensuring co-delivery of a TLR ligand with antigens into the same phagosome was superior in inducing major histocompatibility complex class II (MHC II)-mediated antigen presentation [8]. This type of ‘associative recognition’ [2] of an antigen with PAMPs that engage TLRs could be exploited in tumor vaccine design. We thus hypothesized that tumor antigens would be favored for presentation to T cells upon introducing flagellin into tumor cells. Indeed, we found that simply co-injecting recombinant flagellin with tumor cells failed at inducing tumor rejection and tumor-specific T cell priming compared to engineering tumor cells to express flagellin [6]. Importantly, in a regimen of treating tumor-bearing mice, co-injection of recombinant flagellin with irradiated tumor cells as a vaccine did not protect mice from subsequent challenge with parental tumor cells, and did not impair tumor progression. Our data can partially explain why some TLR ligands tested as single adjuvants in phase II and III clinical trials have failed at providing significant tumor regression [9].

Our findings indicate that at least two criteria must be met mounting a robust immune response against cancer cells: first, dual targeting of TLRs and NLRs with adjuvants, and second, ensuring their delivery with tumor cells to the same subcellular compartments within DC such that tumor antigens may be processed for optimal loading onto MHC molecules. These measures should hold paramount significance in the design of effective future cancer vaccines.
